# How Feasible Is Baby-Led Weaning as an Approach to Infant Feeding? A Review of the Evidence

**DOI:** 10.3390/nu4111575

**Published:** 2012-11-02

**Authors:** Sonya L. Cameron, Anne-Louise M. Heath, Rachael W. Taylor

**Affiliations:** 1 Department of Human Nutrition, University of Otago, Dunedin 9054, New Zealand; Email: sonya.cameron@otago.ac.nz (S.L.C.); anne-louise.heath@otago.ac.nz (A.-L.M.H.); 2 Department of Medicine, University of Otago, Dunedin 9050, New Zealand

**Keywords:** infant, Baby-Led Weaning, breastfeeding, complementary feeding

## Abstract

Baby-Led Weaning (BLW) is an alternative method for introducing complementary foods to infants in which the infant feeds themselves hand-held foods instead of being spoon-fed by an adult. The BLW infant also shares family food and mealtimes and is offered milk (ideally breast milk) on demand until they self-wean. Anecdotal evidence suggests that many parents are choosing this method instead of conventional spoon-feeding of purées. Observational studies suggest that BLW may encourage improved eating patterns and lead to a healthier body weight, although it is not yet clear whether these associations are causal. This review evaluates the literature with respect to the prerequisites for BLW, which we have defined as beginning complementary foods at six months (for safety reasons), and exclusive breastfeeding to six months (to align with WHO infant feeding guidelines); the gross and oral motor skills required for successful and safe self-feeding of whole foods from six months; and the practicalities of family meals and continued breastfeeding on demand. Baby-Led Weaning will not suit all infants and families, but it is probably achievable for most. However, ultimately, the feasibility of BLW as an approach to infant feeding can only be determined in a randomized controlled trial. Given the popularity of BLW amongst parents, such a study is urgently needed.

## 1. Introduction

Advice on infant feeding has changed significantly over the last decade, but typically involved spoon-feeding puréed food from around 4–5 months of age [1]. In 2002, the World Health Organisation (WHO) changed their infant feeding guideline by extending the recommended duration of exclusive breastfeeding from 4–6 months to 6 months [2]. In turn, this meant that the recommended age for starting complementary foods was also increased to six months [3]. Although several countries have adopted these recommendations into policy including New Zealand [4] and the United Kingdom [5], others have yet to do so [6]. Given the considerable differences in development between four and six month old infants, the question arises: is spoon-feeding purées still the best way to introduce complementary foods now that infants are theoretically so much older when solid foods are first introduced. 

In the past, when babies started complementary foods at four months of age, they had to be given purées because they were too young to feed themselves. However, advocates of Baby-Led Weaning (BLW) propose that at six months of age infants are developmentally more advanced and therefore do not require purées or indeed need to be fed by someone else [7]. In fact, some suggest that not assessing the appropriateness of spoon-feeding purées for infants at this new developmental age was an oversight when the WHO recommendations were changed [8].

Baby-Led Weaning is an alternative method of infant feeding which promotes infant self-feeding from six months, instead of conventional parent spoon-feeding [7]. Baby-Led Weaning can be described as having two phases: preparation for BLW from birth to approximately six months, followed by implementation of BLW from around six months onwards. During the preparation phase, infants are ideally exclusively breastfed (although formula or mixed feeding is also possible) and parents wait until the child shows developmental signs of readiness to self-feed at around six months of age [7]. When the infant makes the transition from solely milk to a diet that includes solid, ideally “family”, food, the food is offered as “graspable” pieces and the infant learns to feed themselves [7]. 

Anecdotal evidence suggests that BLW is becoming popular among parents particularly in the United Kingdom and New Zealand. There are numerous internet sites, blogs, and online forums enthusiastically sharing knowledge of and experiences with BLW [9,10,11]. However, there are many unanswered questions, and whether BLW is a useful approach to infant feeding at the population level is unknown. 

This review evaluates the literature regarding how feasible BLW might be for parents in the general population by answering four questions:

1. What is Baby-Led Weaning and when should it begin?
2. Can parents wait until six months to introduce solid food to their infant?
3. Can infants self-feed successfully from six months of age and is it safe for them to eat unmodified family foods this early?
4. Can parents meet expectations around family meals and continued breastfeeding?

## 2. Search Methods

Studies were identified using the electronic databases MEDLINE (1996–20 July 2012), CINAHL (1981–20 July 2012) and Web of Science (1899–the week of 20 July 2012). Table 1 outlines the search strategies and key terms used. Further studies were found using the reference lists of the identified studies. Studies were only included if they met the following criteria:

1. published in English;
2. conducted in an industrialized country (for the purposes of this review: Australia, European countries, New Zealand, United Kingdom, United States of America), *i.e.*, in order to discriminate from countries with a lower standard of living and poorer health status which would complicate the cross-country comparisons of infant feeding practices;
3. reported original data;
4. reported multivariate analysis (only applies to complementary feeding studies);
5. published from 2002 onwards (*i.e.*, since the WHO changed its infant feeding guidelines).

**Table 1:** Search strategies and terms used to identify studies for this review.

Search terms used to identify Baby-Led Weaning studies
baby-led.mp self-feeding.mp finger food.mp family food.mp (1) OR (2) OR (3) OR (4) infant/ feeding method/ (6) AND (7) (5) AND (8)
**Search terms used to identify factors associated with introduction of complementary foods at 6 months**
weaning ventilator weaning (1) NOT (2) 6-month$.mp six-month$.mp six month$.mp 6 month$.mp (4) OR (5) OR (6) OR (7) age factors/ educational status/ home childbirth/ mothers/ time factors/ attitude to health/ intention/ self efficacy/ social support/ socioeconomic factors/ family characteristics/ smoking/ pregnancy/ infant/ (9) OR (10) OR (11) OR (12) OR (13) OR (14) OR (15) OR (16) OR (17) OR (18) OR (19) OR (20) OR (21) OR (22) (3) AND (8) (3) AND (23)

## 3. What Is Baby-Led Weaning and When Should It Begin?

The first research on BLW was a very small observational study assessing five infants’ response to being offered “graspable” pieces of whole food that allowed self-feeding whilst joining in at the family meal [12]. This unpublished Masters work suggested that six month old infants have the necessary motor skills to self-feed pieces of whole food and the author concluded that instead of spoon-feeding, parents should allow infants to “lead the way” by feeding themselves [12]. Publication of a book for the general public advocating BLW as an alternative approach to complementary feeding followed [7]. Although the authors point out that they did not “invent” infant self-feeding because some parents have always allowed their infant to feed themselves for reasons of convenience, or because their infant refused to be fed, by coining the phrase “Baby-Led Weaning” and describing its characteristics the authors have made this approach to complementary feeding accessible to a far wider audience [9,10]. While there is nothing revolutionary about giving six month old infants finger foods, and the UK recommendations suggest that finger foods are introduced from six months of age [13], what separates BLW from conventional feeding is that the baby only has finger foods, making purées and spoon-feeding obsolete [7]. 

Generally with BLW, the infant is offered pieces of whole food in a size and shape that they can pick up and feed themselves, typically those that are “stick-shaped” [7]. The parent decides what to offer but it is the baby who decides what they will eat (of the choices provided), how much and how quickly. Baby-Led Weaning also differs from conventional methods for introducing complementary food in that a wider range of foods are suggested as first foods, including: fruit, vegetables, meat, cheese, well-cooked eggs, bread (or toast), pasta, and most fish [7]. Almost all foods that are offered to spoon-fed infants can be prepared in ways that are appropriate for BLW, although it is less likely that infant cereal would be offered because it cannot be easily picked up with the hands. Table 2 outlines some examples of foods prepared for conventional spoon-feeding that can be adapted for use with a BLW approach. 

**Table 2:** Examples of foods that can be spoon-fed and the equivalent Baby-Led Weaning option.

Food	Conventional method	BLW at age 6 to 7 months
Broccoli	Puréed or mashed	Served as a floret-sized piece, large enough for the infant to hold with some protruding from the fist. Steamed to a soft consistency.
Banana	Puréed or mashed	Skin is left on the bottom section of the banana (this gives the infant something to grip) and the top section is peeled for infant to eat.
Pasta	Puréed with meat or vegetables	Large pieces such as spirals or strips of lasagne are offered as part of the meal.
Beef	Puréed with liquid	Slow cooked or stewed, offered as a chunk or a strip of meat, large enough for the infant to hold with some protruding from the fist.

Proponents of the Baby-Led approach to complementary feeding argue that it is more closely aligned with the self-feeding characteristics of breastfeeding [7]. A breastfed infant is better able to self-regulate their intake by feeding for as long and as often as they need, in contrast to a bottle fed infant who is offered a set amount of milk predetermined by the caregiver, and is therefore a more passive participant [14,15]. In addition, because breast milk changes in flavour according to the mother’s diet, the breastfed infant is exposed to a variety of flavours which prepares them for complementary foods [7]. Exclusive breastfeeding to six months also aligns with WHO recommendations for infant feeding before complementary foods are introduced [2]. The ideal preparation for BLW is therefore exclusive breastfeeding to six months, although Rapley and Murkett [7] suggest that a Baby-Led approach should be possible if the baby is breast or formula- or mixed-fed.

### 3.1. How Is BLW Defined?

One of the difficulties in this area of research is that what constitutes BLW has not been defined. As Table 2 outlines, most existing studies have recruited participants who self-identify as following a BLW approach [16,17,18,19,20,21], with all of the ambiguity that this entails. Only the work of Brown and colleagues [16,17,18] uses a more distinct definition to discriminate between those following a Baby-Led approach and those using more conventional feeding, by asking parents to estimate the proportion of food that is provided as purées or spoon-fed. It is not clear whether a “true” Baby-Led approach includes limited use of purées and spoon-feeding (less than 10%) as defined by Brown and Lee [16,17,18], or a more strict definition where only finger foods are provided [7]. Examination of popular websites dedicated to this topic, and work from our own group [22] would suggest that both views exist among parents who believe themselves to be following a Baby-Led style of infant feeding. 

### 3.2. What Do We Know to Date?

Despite considerable interest in BLW from parents and health workers worldwide, very little research has examined this style of infant feeding (Table 3). What we do know is that parents who follow a BLW philosophy may be different: BLW mothers are more likely to breastfeed, have more years of education, and are less likely to return to work before 12 months postpartum than other mothers [18]. Following a Baby-Led approach to weaning has also been identified as the strongest predictor of weaning (introduction of complementary foods) at the recommended age [21]. Interestingly, parents report choosing BLW because they perceive that it provides a range of potential benefits, including being a healthier, less expensive way of introducing complementary foods, which they perceive baby enjoys more [17,22]. These views contrast to those expressed by some healthcare professionals. Although healthcare professionals did acknowledge there could be benefits of BLW, including promotion of self-regulation of energy intake, their principal attitude was one of concern about the potential increased risk of choking, iron deficiency, and inadequate energy intake. It should be acknowledged however that few had direct experience with BLW; and that their views were in considerable contrast to those of parents who have successfully used this style of feeding who reported no major concerns with BLW and would strongly recommend this feeding approach to other parents [22].

**Table 3:** Studies examining Baby-Led Weaning.

Author (Date) [reference]	Participants	Design and Methods	Definition of BLW	Main findings
Brown and Lee (2011) [18]	*n* = 655	Cross sectional	Spoon-feeding and purée use ≤10% of time	Mothers using BLW had higher education, were more likely to breastfeed and were less likely to be returning to work before 12 months postpartum.
	UK mothers with infant aged 6-12 months	Online questionnaire		
	Recruited online and from community groups			Infants following BLW were more likely to have meals with family and eat the same food as family.
Brown and Lee (2011) [16]	*n* = 652	Cross sectional	Spoon-feeding and purée use ≤10% of time	Mothers following BLW reported lower levels of restriction, pressure to eat, monitoring and concern over child weight compared to mothers following SW.
	UK mothers with infant aged 6-12 months	Online questionnaire		
	Recruited online and from community groups			No association between weaning style (SW or BLW) and infant weight.
Brown and Lee (2011) [17]	*n* = 36	Cross sectional	Self-reported	Mothers reported:
	UK mothers following BLW with infant aged 12-18 months	Semi-structured face-to-face interviews		(1) Positive experiences including: more convenient (at meal times and when out and about), reduced cost, didn’t have to worry about following a plan, thought baby would develop healthier eating patterns, thought baby enjoyed it more and “it made sense”.
	Recruited online at BLW websites			
				(2) Infants participated in family meals and generally ate what the family ate.
				(3) Some challenges including mess, food wastage, and anxiety about potential choking in the first few weeks of BLW.
Rowan and Harris (2012) [20]	*n* = 10	Cross-sectional	Planned to use BLW techniques and had read the BLW book by Rapley and Murkett ∗	Parents offered 57% of family foods to infant.
	Parents of infant aged 6 months	Two 3-DDR at 6 and 9 months		No change in parents’ diets.
	Recruited at BLW websites			
Townsend and Pitchford (2012) [19]	*n* = 155	Case-control	Self-reported	Compared to the SW group, the BLW group demonstrated significantly increased liking for “carbohydrates”.
	UK parent of infant aged (20-78 months)	Questionnaire		
				There appeared to be an increased incidence of underweight in the BLW and obesity in the SW group (significance not tested).
	Recruited online at BLW websites (cases) and from laboratory database (controls)			
Moore, Milligan and Goff (2012) [21]	*n* = 3607	Cross sectional	Self-reported	“Baby-Led” or “finger foods” weaning approach was the strongest predictor for weaning at or later than 26 weeks.
	UK parents	Online questionnaire		
	Recruited at parenting groups and online forums			
Cameron, Heath and Taylor (2012) [22]	Healthcare professionals (*n *= 31)	Cross-Sectional	Self reported	Health professionals suggested potential benefits of BLW such as greater opportunity for family meals, fewer mealtime battles, healthier eating behaviours, greater convenience, and possible developmental advantages. However they also had concerns about potential choking, iron intake and growth.
		Semi-structured interview		
	Mothers who had used BLW (*n *= 20)			
	Recruited by advertisement, email and parenting groups			
				Mothers considered BLW to be a healthier, more convenient and less stressful way to introduce complementary foods.30% (*n* = 4) mothers reported at least one choking episode-most commonly with raw apple.

BLW, Baby-Led Weaning; SW, standard weaning (spoon-feeding purées); 3-DDR, three day diet record; ∗ [7]; GMS, Gateshead Millennium Study.

It has been suggested that BLW may encourage greater acceptance of foods with a variety of textures and flavours and that this may result in higher intakes of “healthier” foods such as vegetables and unprocessed foods as the child grows. Furthermore, the fact that eating is more under the control of the infant in a Baby-Led approach has interesting implications for the development of appropriate energy self-regulation and the development of obesity [7]. Certainly the observation that breast-fed babies have improved energy self-regulation at 18–24 months of age [23], provides some support for this hypothesis, given that breastfeeding represents an infant’s first exposure to a Baby-Led style of feeding. However, one of the difficulties in this area is that a Baby-Led approach, by its very nature, also encourages a more responsive eating style, as the term is used in the context of obesity prevention (see Table 4). In BLW, the parent provides the food but the infant is in control of exactly what and how much they eat. The infant is also encouraged to join in at family mealtimes, and the infant is never hurried or forced to eat food. Nonresponsive feeding is thought to override the child’s internal hunger and satiety regulatory cues, causing the child to lose the ability to accurately respond to their own physical hunger signals [24]. A recent review on the development of healthy eating habits early in life found that responsive feeding was one of the most important practices for encouraging healthy eating habits in early life and should be encouraged in parents to reduce the risk of obesity [25]. Little data exists in the context of responsive feeding and BLW. Brown and Lee [16] found in that mothers who followed BLW were less likely to pressure their child to eat and restrict food (two non-responsive feeding practices) than mothers who used more standard weaning practices (spoon-feeding purées). However, no prospective data exists to disentangle whether a BLW approach discourages these adverse feeding practices or whether having these maternal characteristics makes BLW more attractive in the first place.

**Table 4:** Two approaches to responsive feeding.

Responsive Feeding as Defined by Black [24] in the Context of Obesity Prevention	Responsive Feeding Defined by WHO [1] in the Context of Health and Illness
Ensure that the feeding context is pleasant with few distractions; that the child is seated comfortably, ideally facing others; that expectations are communicated clearly; and that the food is healthy, tasty, developmentally appropriate, and offered on a predictable schedule so the child is likely to be hungry. Encourage and attend to the child’s signals of hunger and satiety. Respond to the child in a prompt, emotionally supportive, contingent, and developmentally appropriate manner.	Feed infants directly and assist older children when they feed themselves. Feed slowly and patiently, and encourage children to eat, but do not force them. If children refuse many foods, experiment with different food combinations, tastes, textures and methods of encouragement. Minimize distractions during meals if the child loses interest easily. Remember that feeding times are periods of learning and love—talk to children during feeding, with eye-to-eye contact.

Similarly, it is interesting to speculate whether infant temperament affects the success of BLW in that an “easy” baby may respond to a Baby-Led approach that allows exploration of food. To date, no studies have examined temperament in relation to BLW, although it is known that “fussy” babies are introduced to complementary foods earlier than their counterparts [26]. Only one small study has examined eating patterns and body mass index (BMI) in Baby-Led weaned infants. Overall, those in the BLW group had healthier dietary intakes as indicated by an increased liking for carbohydrate foods found at the bottom of the food pyramid compared to the spoon-fed group who preferred sweet foods. Although a lower prevalence of overweight was also reported in the BLW group, insufficient sample size precluded statistical examination of this observation [19]. 

In a world where childhood obesity rates are climbing [27] BLW may offer an alternative method for introducing complementary foods that encourages the use of responsive feeding principles, which many in turn result in healthier weight gain. This interesting theory has not, however, been tested. If BLW is associated with healthier body weight at this age, only a randomized controlled trial would be able to ascertain whether this was due to infant self-feeding (*i.e.*, BLW), or perhaps the result of other factors such as socioeconomic status and maternal education, both of which have been associated with BLW [18], and with body weight in infants who are not BLW [28].

### 3.3. At What Age Should BLW Commence?

In its purest form, the implementation of BLW begins at around six months when the infant is developmentally able to self-feed as indicated by the ability to sit independently and rake, scoop, or hold food and bring it to their mouth [7]. The development of these motor skills and the need to be sitting upright for safety reasons is outlined in more detail in section 5. However, commencing BLW at six months of age also aligns with current WHO recommendations regarding complementary feeding [29].

The overarching principles of BLW are therefore: the infant is milk fed, ideally exclusively breastfed, until approximately six months of age, they then transition to pieces of whole food that they feed themselves, and the infant shares their meals with the family, eating the same food as the family wherever possible. Breastfeeding (or formula feeding) continues on demand throughout BLW until the infant chooses to wean themselves completely from breast milk.

## 4. Can Parents Wait until Six Months to Introduce Solid Food to Their Infant?

Following breastfeeding (or formula feeding), complementary feeding constitutes the next major feeding stage in an infant’s life. It corresponds to two particular changes: the decline and eventual cessation of milk feeding, and the progressive introduction of culturally accepted family foods [30]. In this review the term “complementary feeding” refers to all solid and liquid foods other than breast milk or infant formula. Other terms commonly used in this context are “solids”, “weaning”, “weaning foods”, and “beikost”. The WHO states that “any nutrient-containing foods or liquids other than breast milk given to young children during the period of complementary feeding are defined as complementary foods” [29]. The WHO emphasizes that complementary feeding should be timely (starting at six months of age (180 days)), safe, adequate in terms of variety, frequency, amounts and consistency, and that complementary foods should be offered in a suitable way that is relevant to the infant’s development [29]. Prior to 2002, the WHO recommendation was that complementary food could begin at 4–6 months of age. In 2002, this recommendation was changed to six months of age. However there is very little research specifically on the age of introduction of complementary food, with greater emphasis having been placed on the duration and predictors of exclusive breastfeeding (the one being closely related to the other, at least for breastfeeding mothers), and most of the research still reflects the old recommendation (introduction at 4–6 months). 

Data on the age of introduction of complementary foods do not exist for many countries, including New Zealand. Data from the 2001 Australian National Health Survey showed that 55% of Australian infants were receiving complementary food at 18 weeks of age (~4.5 months) [31]. A similar proportion of UK mothers (51%) had introduced complementary foods by four months in 2005 [32]. Data from most other countries highlight a discrepancy between the WHO recommendations and actual practice, demonstrating that many mothers do not follow this recommendation [33,34]. However as is the case with exclusive breastfeeding to six months, national recommendations in many countries have only recently moved the age for introduction of complementary foods to six months. Therefore it is yet to be seen whether the new recommendation, given time and promotion, will be adhered to. 

Although data are very limited, it appears that mothers who choose to follow BLW may be more likely to meet the WHO recommendation for beginning complementary feeding at six months. In a group of 36 women following BLW, the average age for starting complementary foods was 25 weeks (range: 22–32 weeks) which is just short of the WHO recommended six months (26 weeks) [17]. Furthermore, 55% of participants reported waiting until six months to start [17]. A much larger internet-based survey in more than 3600 UK parents showed that using a “Baby-Led” or “finger foods” weaning approach was the most reliable factor in predicting the probability of weaning at or later than 26 weeks [21]. However, neither of these cross-sectional studies is able to elucidate whether planning to follow BLW has an impact on the timing of starting complementary foods. It is not clear whether BLW leads to later initiation of complementary feeding, or whether parents who manage to wait until six months of age choose BLW as the method of complementary feeding.

### 4.1. Risk Factors for Introducing Complementary Foods before Six Months

It is obvious that the majority of parents do not achieve the WHO recommendation to wait until six months to begin complementary foods. To determine the extent to which it is possible to improve these rates it is necessary to identify the factors associated with later introduction of complementary food. Although the “new” WHO recommendations have been in existence for 10 years, research still generally reflects the previous recommendation that complementary foods are started at four to six months of age. All papers referred to in this section therefore compare early (usually defined as before four months) with later (after four months) introduction.

For the purposes of this review, factors influencing delaying the introduction of complementary foods have been broadly categorized as maternal and infant variables. The maternal variables that are discussed are: age, education, ethnicity, short duration or lack of breastfeeding, socioeconomic status, BMI, parity, psychology, beliefs and smoking. The infant variables that are discussed are allergic predisposition, birth weight, sex, temperament and childcare. Table 5 summarises the papers discussed under these headings.

**Table 5:** Factors associated with the introduction of complementary foods.

Author, year [reference] country	*N*	Study Type and Methods	PositiveAssociation	Negative Association	No Association	Confounders Adjustedfor
Alder *et al. *(2004) [35] UK	286	Prospective Cohort		Influenced by grandmother’s opinion, high deprivation score, disagree with IFR, friends disagree with IFR, received free sample of baby food	Maternal age, infant sex, employed before pregnancy, looking forward to giving solids, giving the infant solids <4 months means they have reached a milestone, people who are important to me say wait until 4 months	Maternal age, infant sex, influenced by grandmother’s opinion, high deprivation score, disagree with IFR, friends disagree with IFR, received free sample of baby food, employed before pregnancy, looking forward to giving solids, giving the infant solids <4 months means they have reached a milestone, influence of others
		Interview at 12 weeks, & Questionnaire at 20 weeks postpartum				
Coleman *et al.* * (2009) [36] Canada	2153	Cross sectional		Maternal smoking during pregnancy, FF infant, living with a smoker, maternal age (≤27 years) not attending prenatal class, low, first child	Parity	Maternal age, maternal smoking during pregnancy, parity, marital status, method of infant feeding at 3 months, living with a smoker, prenatal class attendance, income
		Phone survey at 3 & 9 months postpartum				
Crocetti, Dudas and Krugman (2004) [37] USA	102	Cross sectional		Ethnicity (Hispanic)	Maternal age (<20 years), maternal ethnicity, maternal education, BF, Medicaid	Maternal age, caring for 1 child, awareness of infant feeding guidelines, race, insurance, maternal education, type of milk feeding
		Questionnaire at 4 months postpartum				
Dratva, Merten & Ackermann-Liebrich (2006) [38] Switzerland	2868	Cross sectional	Multiparous (Swiss mothers only), allergic predisposition (Swiss mothers only)	Maternal age (<20 years), residing in French & Italian speaking region of Switzerland, high maternal BMI, maternal smoking before birth	Mothers attentiveness to own diet, health problems at birth, health problems now, birth weight	Income, maternal education, work after birth, raising child alone, infant sex, nationality
		Questionnaire & 24 h dietary recall				
Erkkola *et al .*(2005) [39] Finland	429	Prospective cohort	Maternal age (1 year increments), high maternal & paternal education, infant sex (girl)		Not listed	Maternal age, maternal education, infant sex, parity, infant’s ponderal index at birth
		Questionnaire at 3, 6, 12, 18, 24 months postpartum				
Fewtrell *et al.* (2003) [40] UK		Data from >2000 infants from 7 prospective randomised trails	Maternal age	Smoking, not BF	Birth weight	Type of milk fed, maternal age, birth weight, sex, whether mother and father smoked during 2nd and 3rd trimesters, social class, child’s birth order, maternal education
Giovannini *et al.* (2004) [41] Italy	1221	Cross sectional		Introduction of formula, not BF infant, infant weight at 1 month, maternal smoking	Maternal age, pacifier use at 1 month	Maternal age, maternal BMI, maternal education, maternal smoking, type of delivery, mother having been breastfed, infant sex, infant weight at 1 month, pacifier use at hospital ward & at 1 month, parity, introduction of formula, formula promotion at discharge, time of initiation of BF
		National survey				
		Phone Interview at 30 days, 1, 3, 6 & 9 months postpartum				
Griffiths *et al.* (2007) [42] England	11,286	Cross sectional analysis of MCS	Ethnic minority, mother not returning to work ≤4 months postpartum	Low maternal education (non-white), high SES (non-white), stopped BF ≤4 months	Maternal qualification (white), lone mother status,	SES, maternal education, maternal employment status, lone mother status, maternal age at MCS birth, maternal age at first birth, parity, BF
		Questionnaire at 9 months postpartum				
Grummer-Strawn *et al.* (2008) [43] USA	2707	Prospective cohort (IFPS II)	Maternal age (>25 years), high maternal education, living in western region, BF in hospital	WIC participant, Income (185%-350%, of federal poverty level)	Race, parity	Maternal age, education, ethnicity, race, parity, income, WIC participant, region, BF in hospital & BF at 24-28 weeks
		Questionnaire (1-6 months) then 7½, 9, 10½, & 12 months				
Hampson *et al.* (2010) [44] Norway	37,919	Prospective cohort		High negative affectivity score	Not listed	Maternal age, maternal BMI, maternal education
		Questionnaire at 17 & 30 weeks gestation & 6 months postpartum				
Hart & Drotar (2006) [45] USA	98	Cross sectional	Doctor’s recommendations to start food, marital status		Number of solutions generated to child-rearing problems	Marital status, child’s age
		Questionnaire (infant aged 6-18 months)				
Kim *et al. *(2008) [46] USA	8150	Cross sectional		Attending childcare before 3 months of age	Not listed	Infant age, sex, race/ethnicity, birth weight, prematurity, household poverty, maternal education, maternal employment, marital status, maternal smoking, maternal pre-pregnancy BMI, BF initiation or early introduction of food
		Interview at 9 months				
Lande *et al. *(2003) [47] Norway	2383	Cross sectional Nationwide survey	Maternal age (>25), maternal education, degree of urbanization, infant sex (girl), geographic region (east, south, top, north)	Maternal smoking, geographic region (west)	Not listed	Maternal age, maternal education, maternal smoking, degree of urbanization, infant sex, geographic region
		FFQ at 6 months & retrospective FFQ for ≤5 months, birth records				
Rebhan *et al.* (2009) [48] Germany	3103	Prospective cohort		Maternal age (>34 years), low maternal education, country of birth (outside Germany), maternal smoking, not BF at 4 months	Family status, infant sex, parity, BF problems, attitude of father to BF, parents with allergy, caffeine, maternal BMI, districts of Bavaria, clinic size	Maternal age, maternal education, country of birth, maternal smoking, infant sex, BF at 4 months, family status, parity, BF problems, attitude of father to BF, parents with allergy, caffeine, maternal BMI, districts of Bavaria, clinic size
		Questionnaire at 6 days, 2, 4, 6 & 9 months				
Schiess *et al. *(2009) [49] 5 European countries (Germany, Belgium, Italy, Poland, Spain)	851 FF, 349 BF	Multicentre intervention		Low maternal education (FF & BF), maternal smoking (FF), country of residence (Belgium & Spain) for BF, only Belgium for FF	Maternal age (BF), maternal smoking (BF), birth weight, birth order, infant sex	Country of residence, maternal age, maternal education, maternal smoking
		3-DDR at each completed month from 1-9 &12				
Scott *et al. *(2009) [50] Australia	519	Prospective cohort (PIFSII)		Maternal age (<20 years), maternal smoking, fully FF at 4 weeks, partially BF at 4 weeks	Infant sex, birth weight, admission to SCN, infant feeding at discharge, maternal education, marital status, country of birth, parity, mother returning to work at 12 months, infant feeding attitude score	Maternal age, maternal smoking, infant sex, birth weight, infant feeding at 4 weeks, admission to SCN, infant feeding at discharge, maternal education, marital status, country of birth, parity, mother returning to work at 12 months, infant feeding attitude score
		Baseline Qu, phone Intvs at 4, 10, 16, 22, 32, 40 & 52 weeks				
Tatone-Tokuda *et al.* (2009) [51] Canada	2223	Cross-sectional analysis on QLSCD	Low maternal self-efficacy ^‡^, immigrant	Maternal smoking during pregnancy, low maternal education, maternal age (<35 years), parental impact ^‡^, low SES, infant sex (boy), infant birth weight (>2.5 kg)	Mother’s perception of overprotectiveness, main employment status of the mother	All variable in model (maternal self-efficacy, mothers perception of parental impact, mother smoking during pregnancy, maternal education, maternal age immigrant status, SES, infant sex, infant birth weight
		Interviews & Questionnaires at 5 months, birth records				
Tarrant *et al. *(2010) [52] Ireland	401	Prospective cohort	Public health nurse as principal source of advice	Maternal age (≤34), low maternal education, mothers reporting infants should start at 12 weeks, FF at 12 weeks, maternal grandmother as principal source of advice	Smoking status during pregnancy, birth weight	Maternal age, maternal education, smoking status during pregnancy, parity, infant birth weight, gestational age of infant at birth
		Questionnaire & DH at 6 weeks & 6 months				
Wasser *et al. *(2010) [26] USA	217	Cross sectional anaylsis of the Infant Care study	Any BF, maternal education	Distress to limitation, activity level, maternal obesity (BMI ≥ 30)	Soothability, infant sex, infant age, social desirability	Maternal BMI, soothability, infant sex, infant age, social desirability, distress to limitation, activity level
		Infant DH, 24 h DR at 3 months postpartum				
Wright *et al. *(2004) [53] UK	207	MCS	BF at 4 months	Baby seemed hungry, high deprivation score	Not listed	BF at 4 months, baby seemed hungry, high deprivation scores, infant weight gain	
		Questionnaires at 6 weeks, 4, 8, 12 months postpartum				

BF, Breastfeeding; BMI, Body Mass Index; DH, Diet history; DR, Dietary recall; FF, Formula feeding; FFQ, Food frequency questionnaire; IFPS II, Infant Feeding Practices Study II; IFR, Infant feeding recommendation; MCS, Millennium Cohort Study; PIFS II, Perth Infant Feeding Study II; QLSCD, Québec Longitudinal Study of Child Development; SCN, Special care nursery; SES, Socioeconomic Status; WIC, Support for low-income mothers; 3-DDR, Three day diet record; * measured the introduction of cereal only; ^‡^ borderline significant (CI includes 1.0, *p* value significant).

The literature is consistent in demonstrating that younger *maternal age* is associated with introducing complementary foods earlier [36,38,39,40,43,47,48,49,50,51,52] with few exceptions [35,41].

Low *maternal education* increases the risk of introducing complementary foods early [26,43,47,49,50,51,52], with mothers with 10 years or less of education being three times more likely to introduce complementary foods before the infant was five months of age than those with more education [48]. Although some studies have not observed any relationship between education and timing [37,39,42], others [39] have demonstrated that complementary foods were likely to be introduced at the appropriate age only if both parents were highly educated (education level above high school) (OR 0.50; 95% CI 0.29–0.85). 

Few studies [37,42,54] appear to have examined differences in complementary feeding across different *ethnicities*. Two have reported that early introduction of complementary food (before four months) was associated with belonging to an ethnic minority rather than the white majority [42,54], with one also demonstrating an interaction with education [42]. However Crocetti *et al.* [37] reported no association between white ethnicity and age of introduction of complementary foods, and reduced odds amongst those of Hispanic ethnicity. These differences in findings may arise because relatively broad ethnic categories can hide huge variation in life experience and cultural environment across a region or country. This has been highlighted by several groups [38,43,47,49] who have showed that the country or region of residence is linked to the timing of introduction of complementary foods.

It is difficult to tease out the relative importance of factors such as *socioeconomic status *(*SES*), education and ethnicity given the interrelationships between these variables. In addition, socioeconomic status is very difficult to assess and most papers report very few categories (e.g., low SES *vs.* higher SES), which may obscure important social gradients that apply across the entire socioeconomic spectrum [55]. However, it appears that the association between early introduction of complementary foods and SES generally follows a similar pattern to education [35,36,43,51,53]. Participants with a low SES (high deprivation) score were 70% more likely to begin complementary foods before three months in a UK sample [35] and 50% more likely to begin before four months in a Canadian sample [51]. Contrasting this, Griffiths *et al.* observed in the Millennium Cohort Study [42] that early introduction of complementary food was associated with high SES (stratified for ethnicity) for those of non-white ethnicity, although the more commonly reported association was seen in those of white ethnicity. 

Many researchers have identified a strong negative association between *absence or short duration of breastfeeding* and meeting the recommendation for age of introduction of complementary food [26,40,41,43,48,50,52]. Using multivariate analysis, Rebhan and colleagues [48] identified sub-optimal breastfeeding practices as the strongest (over and above maternal age, education, country of birth, smoking, and infant sex) risk factor associated with early introduction of complementary foods (OR 8.57; 95% CI 6.16–11.94). Furthermore, the introduction of formula to replace breast milk was also identified as an important factor associated with early introduction of complementary foods [36,41]. Mothers in Italy who introduced their infant to formula within the first month of life were 2.7 times more likely to start complementary foods early [41]. The findings from Coleman and colleges [36] were similar, showing that infants who were formula fed at four months were more than twice as likely to receive complementary foods early. 

High *maternal BMI* may negatively impact on meeting the recommended age for introducing complementary foods. Overweight or obese mothers have more problems with breastfeeding, so are more likely to stop breastfeeding before six months [56,57], and if they introduce complementary foods in place of breast milk will be introducing complementary food early. In the literature, two studies have reported a significant association between BMI and timing of introduction of complementary foods [26,38], and one reported no association [48]. In one Swiss study, overweight (BMI 26–30), but not obese (BMI > 30), mothers were more likely to start complementary foods early than mothers in the healthy weight range (BMI 19–25) [38]. However, wide confidence intervals in the obese group suggest that more participants were needed and this may be why no association was seen in this group. 

*Parity* does not appear to impact on the timing of complementary foods [36,43,48,50], with the exception of a single study demonstrating that the presence of siblings was protective for Swiss women but not for non-Swiss women [38]. 

Aspects of *maternal psychology* can impact on timing of introduction of complementary foods. Mothers with negative affectivity (high anxiety and or depression) have been reported to be 30% more likely to introduce complementary foods before three months [44]. Furthermore, mothers who believed they had little impact as a parent may be more likely to start complementary foods early (OR 1.4; 95% CI 1.0–1.8) [51]. 

*Maternal beliefs* are shaped by social, cultural and family interactions. In many cultures the influence of a family member or significant other is more influential than the advice of a healthcare professional [58]. Furthermore, multigenerational households can be beneficial for support but can also be a source of immense tension as mothers and grandmothers struggle to define their roles in caregiving activities, including feeding [59]. The strong influence of cultural and familial beliefs passed down between generations is one plausible reason for the substantial delay in infant feeding practices changing to reflect contemporary recommendations [58]. Often cultural norms promote the early introduction of complementary foods as a strategy to manage the infant’s perceived hunger, or to settle the infant and promote sleep. Two studies have identified that being influenced by the maternal grandmother [35] or using them as the principal source of infant feeding advice [52] was associated with an increased risk of starting complementary food before 12 weeks. By contrast, using the public health nurse as the main source of information was associated with later introduction of complementary foods [52]. 

Wright *et al.* [53] found that perceiving the *infant was hungry* was an independent predictor for beginning complementary foods before 13 weeks. Mothers who did not agree with the recommendation to introduce complementary foods at four months (the recommendation at the time these data were collected) were also more likely to start complementary foods before 12 weeks [35]. Some studies have documented the main reasons reported by mothers for starting complementary food, and although these are descriptive rather than quantitative they do add to our understanding of why parents decide to introduce complementary foods early. The most common reasons reported are that the infant was hungry and milk was no longer enough to satisfy them [37,50,52], to help the infant sleep better at night [37,50,52], advice from a family member or friend [52], or the perception that the infant was a “big baby” and therefore needed more [52]. 

There is no doubt that *smoking* is negatively associated with the timely introduction of complementary foods [36,38,40,41,47,48,49,50,51]. A German study by Rebhan and colleagues [48] reported that after adjusting for a number of variables, even smoking as few as 1–5 cigarettes per day was associated with a doubling of the risk of starting complementary foods early, with the odds increasing 3 fold if more than five were smoked per day. 

The number of infants diagnosed with an *allergy* has increased in the last 10 years and infant allergies can be very difficult for parents to manage [60]. The evidence surrounding the timing of complementary foods and risk of allergies remains controversial and for many reasons, including poor study design and varying outcome measures, it is difficult to disentangle the relationship [61,62,63,64]. One study [38] assessed the perceived risk of allergies and the timing of introduction of complementary foods in families where allergies were already present. These mothers believed that exclusive breastfeeding and the timely introduction of complementary foods reduced the risk of allergy, and were less likely to introduce complementary foods early. However, Rebhan and colleagues [48] found that allergies present in the parents did not affect the timing of introduction to complementary foods. 

Whether *birth weight* is related to the timing of complementary feeding appears more controversial. While some studies have shown that greater weight at birth or one month [41] was significantly associated with the early introduction of complementary food, others [38] including three prospective cohort studies in Ireland [52], Australia [50] and a multi-country analysis [49] found no association between early introduction of complementary food and birth weight. It is possible that this discrepancy occurs as a result of variation in adjustment for confounders; Fewtrell *et al*. [40] demonstrate that the association between birth weight and timing of complementary foods disappears once maternal BMI is removed from the model [40]. This suggests that infant birth weight and early introduction of complementary foods may both be influenced by maternal BMI, rather than being directly related to each other. 

*Infant sex* was associated with the timing of complementary food introduction in some [39,47,51] but not all [35,48,49,50] studies. In studies reporting an association, mothers were 44%–60% more likely to begin complementary foods early with boys compared to girls [39,47,51] and it is thought this practice may be due to mothers perceiving that “boys need more” [65]. 

*Infant temperament* also appears to influence the timing of complementary foods. “Fussy” infants were nearly twice as likely to receive complementary foods before four months in one study [26]. *Childcare* has also been associated with the early introduction of complementary foods [46]. US infants who were in childcare before 3 months were 1.7 times more likely to have started complementary foods before four months of age [46].

In summary, maternal education appears to be positively associated with introducing complementary foods at the appropriate age. In contrast, short duration or lack of breastfeeding, certain maternal beliefs, and maternal smoking are associated with early (before four months) introduction of complementary foods. For all other factors discussed (maternal age, socioeconomic status, ethnicity, BMI, parity and psychology; and infant allergic predisposition, birth weight, sex, temperament and childcare) more research is needed, either because of conflicting results or limited evidence, or because further adjustment for confounders is required.

### 4.2. How Modifiable Are These Risk Factors?

Some factors associated with early introduction of complementary foods are modifiable and interventions to address these factors could result in moving global rates closer to the WHO recommendation while also giving parents the option of transitioning to either conventional spoon-feeding or BLW at six months. Some studies have had great success in modifying factors associated with the early introduction to complementary foods in specific populations [66,67]. These interventions have provided personalized education sessions around the WHO recommendation, the importance of timely introduction to complementary food, and how to appropriately identify and respond to infant cues. These interventions resulted in significantly fewer infants being introduced to complementary foods before four months compared to the control group (23% *cf.* 41% [66] and 57% *cf.* 82% [67]). However, these studies assessed “early introduction” at four months rather than at six months. It is yet to be determined if interventions can successfully delay the introduction of complementary foods until six months of age. A randomized controlled trial is currently underway in New Zealand with one aspect of the intervention being to delay the introduction of complementary foods to six months [68].

### 4.3. Implications of Waiting until Six Months to Introduce Complementary Foods

Ideally infants would be exclusively breastfed until they reached six months of age and were developmentally ready to begin BLW. “Ideally” because there are health benefits of exclusive breastfeeding to six months for both the infant, and the mother [2]. However, at a global level, most women still cease exclusive breastfeeding well before six months. Major methodological issues including the quality of the data sets, lack of adjustment for confounders, not distinguishing between “any” or “exclusive” breastfeeding, and differences in the definition used for “exclusive” breastfeeding make cross-country comparisons of either the prevalence or predictors of exclusive breastfeeding to six months problematic [69,70]. 

Nevertheless, the proportion of women who manage to exclusively breastfeed to six months (for the countries defined in this review) appears to be very low—between 7% [47] and 23% [71]. In many countries, there has been a time lag between changes to the WHO recommendations (2002) and adoption of these changes at national policy level. Although promotion initiatives appear to have increased the proportion of women managing to exclusively breastfeed to six months [71,72], it is likely that even with intensive promotion a substantial number of women will not achieve exclusive breastfeeding to six months, presumably reflecting difficulties mothers face in achieving exclusive breastfeeding for this prolonged period. 

It is not clear what the rates of exclusive breastfeeding are in mothers who follow BLW because BLW is a relatively recently defined method of infant feeding and no studies have directly compared breastfeeding rates in women following BLW with rates in those using more conventional complementary feeding. However, a few observational studies [17,18,19] have detailed the breastfeeding practices of women who have followed BLW. Compared with national exclusive breastfeeding rates, mothers following BLW appear to exclusively breastfeed for longer, with exclusive breastfeeding durations ranging from 18 to 32 weeks [17,18,19]. However, many of the women following BLW still do not reach the recommended six months (26 weeks) of exclusive breastfeeding. There are also some limitations to these studies, in that they are all retrospective and the participants were not typical members of the general population—the majority being educated women, aged 25 years or older. It is therefore hard to determine whether intending to follow BLW is associated with a longer duration of exclusive breastfeeding, or whether both longer duration of exclusive breastfeeding and following BLW are more likely in older more highly educated women. It also seems feasible that mothers who breastfeed for longer are attracted to BLW. They are used to following a Baby-Led feeding pattern already and may conceivably have lower anxiety about monitoring and controlling infant food intake. It could also be seen as the “right” thing to do just as exclusive breastfeeding is the right thing to do in the current parenting climate in countries such as New Zealand and the UK. 

Mothers who cannot breastfeed, or who choose to cease before six months, may choose to formula feed their infant. Although BLW does not preclude the use of formula [7], it is possible that the introduction of formula may hinder the transition to BLW. Formula feeding does not offer the same flavour variation as breast milk. It is the sensory properties of breast milk that are thought to facilitate the transition to the modified adult diet because many flavours from the maternal diet appear in breast milk, promoting the acceptance of a variety of flavours [73], as long as the mother regularly eats the food herself [74,75]. In contrast, formula provides the infant with the same consistent flavour experience. Formula feeding may also override the self-regulation of food intake that BLW attempts to maintain [14,15]. Self-regulation of breastmilk intake appears to be innate in infancy with infants eating fairly accurately in response to their internal hunger and satiety cues [76]. Poor energy self-regulation in older age groups has been associated with the development of overweight and obesity [77]. When infants are bottle-fed they can obtain milk with less effort than from the breast, so the formula-fed infant is more passive in the feeding process making it easy to over-feed [15]. In contrast, the breastfed infant must take an active role in order to transfer milk from the breast. The higher levels of maternal control that are possible with bottle-feeding also reduce the infant’s opportunities to control the amount consumed at a meal and as a result they are likely to learn to empty the bottle instead of responding to their internal cues [14,76]. The extent to which the type of milk fed in the first six months impacts on the success of BLW has not, however, been investigated.

### 4.4. What If Parents Can’t Wait until 6 Months to Introduce Complementary Foods?

Families who cannot wait until six months before introducing food do not have the option to begin BLW early. Studies detailing infant development have established that infants are not ready for food (puréed or finger foods) before four months [78], and as discussed below, the motor skills needed for BLW only develop (in the majority of infants) at six months [78,79]. This then raises the dilemma—what do parents do if they want to follow a Baby-Led approach, but for whatever reason, cannot wait until the infant is six months of age. The only safe feeding options for infants stopping exclusive breastfeeding before six months are the introduction of formula or the conventional method of spoon-feeding purées. It is not clear from the limited literature available whether BLW mothers move their infant to infant formula, spoon-feed purées, or commence a BLW approach earlier than six months of age if they are stopping exclusive breastfeeding early. It may be an option to transition from purées to BLW once the infant reaches six months. However, this does not appear to be the optimal practice advocated by BLW proponents and we are unable to tell if this would have the same potential benefits as BLW alone, in the absence of studies looking at this issue. In practice, the majority of parents wanting to follow a Baby-Led approach to complementary feeding are going to have to use a breast milk substitute or purées at some point given that rates of exclusive breastfeeding to six months are uniformly low across the world [71,72,80,81,82,83].

## 5. Can Infants Self-Feed Successfully from Six Months of Age, and Is It Safe for Them to Eat Unmodified Family Foods This Early?

Assuming the infant arrives exclusively milk fed to six months, there are a number of questions that need to be answered for BLW to be safe. Does a six month old infant have the necessary motor skills to pick up food? Do they have sufficient physical stamina to feed themselves enough food to keep pace with their rapid growth? Is their oral motor function sufficiently developed or will they be at increased risk of choking? Will energy and nutrient intake be adequate? And are family foods always appropriate foods for infants? 

The acquisition of feeding skills has been discussed in the literature with the consensus being that normal healthy infants will develop the skills for self-feeding around six months of age [78,79]. The *motor skills* required for self-feeding are postural stability to sit with little or no help, and to reach for and grasp objects [78,84]. Early work [85,86] found that the emergence of self-sitting is one of the first major milestones of motor development and occurs around five months of age. A more recent study in the USA [87] also demonstrated that infants could sit in their caregiver’s lap without help at an average of five and a half months of age. Self-sitting is necessary for successful self-feeding because once the infant has mastered the ability to sit with little or no support their arms are free to reach for food, instead of being used for balance [85,86]. Interestingly, at the time self-sitting abilities emerge (around 5 months), infants also start to develop coordinated use of their hands in object manipulation and exploration [88]. They also begin to discern between object size and physical properties and will adjust their reach to suit [89]. Two studies have used representative samples to assess the ability of infants to reach out for food as reported by parents [84,90]. It is interesting to note that these data were collected before BLW became popular and before the WHO recommendations for introducing complementary food changed to around six months of age. In other words the majority of parents were spoon-feeding purées and starting complementary foods at four months. The percentage of children who were able to grasp food with their hands was 68% by 4 to 6 months [84], 85% by 6 to 7 months [90] and 96% by 7 to 8 months [84]. However, Wright and colleagues [90] point out that the number of opportunities to reach out for food were significantly greater for infants who reached out for food earlier than for those who reached out later. These authors concluded that parents who had low expectations of their infant’s self-feeding abilities, or who perceived their infant should be spoon-fed purées, were less likely to offer whole foods and as a consequence their infant could not display and practice their self-feeding skills.

The motor skills that emerge around six months of age seem to allow the majority of infants to reach out and grasp food, and, based on the observational studies, it seems reasonable to expect that the majority of (although not all) infants could cope with self-feeding at six months. 

Alongside the motor skills required for successful self-feeding an infant must have the *physical stamina and *“*interest*” in eating to consume enough energy to keep pace with their needs for rapid growth. If they do not, they may be at risk of inadequate energy and nutrient intake, and consequently failure to thrive (growth faltering). Failure to thrive is defined by WHO as a current weight or rate of weight gain that is significantly lower (<−1 SD score) than that of other children of similar age and sex [91,92]. The literature suggests there is an array of reasons (organic and inorganic) for failure to thrive, but problems related to oral and motor function have been identified as a common contributing factor [93,94]. Currently no large well-designed study has investigated the risk of failure to thrive in infants following BLW, although one small study suggests that it may be an issue for some infants [19]. It is uncertain whether an infant’s physical stamina could impact on their self-feeding ability, or energy intake. At greatest risk of failure to thrive would be infants whose self-feeding skills are less than optimal and who are left to their own devices, receiving no assistance from their parents. Although assistance from parents is not encouraged in BLW, some flexibility may be required for infants with poorer self-feeding skills.

Wright and colleagues [90] showed that children with failure to thrive (growth faltering) were later to start finger foods (7.2 months) compared to controls (6.1 months). However the case-control nature of this study does not allow us to determine causality. Failure to thrive may have resulted from the late transition to finger foods, or preceding under-nutrition could have resulted in developmental delay and lack of energy to self-feed finger foods. Furthermore, children who were later to reach out for food, were also later to achieve other developmental milestones [95]. The findings from Carruth and colleagues [84] that successful self-feeders had higher nutrient intakes at 9–11 months of age than those who were not self-feeding, suggests that children following this style of feeding are likely to be consuming sufficient energy. However, whether this applies earlier in the complementary feeding period is unknown given that no studies have directly measured energy and nutrient intakes of children following a Baby-Led approach. 

The period from birth to two years of age is the peak age not only for growth faltering, but also for common *childhood illnesses* [3]. One NZ study showed that 12–24 months old infants on average had 13 separate “unwell” events per child per year [96]. Others [97] have also shown high rates of diarrhea and colds in young children. During a period of illness infants may experience decreased appetite as well as decreased stamina for self-feeding. The WHO recommends “increasing fluid, including more frequent breastfeeding, and encouraging the child to eat soft, varied, appetizing, favourite foods” in times of illness [1]. The WHO emphasises that applying the principles of responsive feeding is equally as important as the types of food offered [1]. The term “responsive feeding” in this sense is used to describe caregiving that applies the principles of psychosocial care (summarized in Table 4). For parents following BLW it may be particularly important to follow the WHO principles of responsive feeding at times of illness in order to maintain nutrient intake. It is likely that BLW may require some modification during times of illness and slightly more intervention from the parent at least until the infant is completely well again. For example, it is possible that some spoon-feeding might be required if a parent felt that the child was not receiving sufficient energy during times of illness. However, whether “encouraging” the child to eat is sufficient, or a more active style of feeding is necessary has not yet been determined. 

Once an infant masters the ability to pick up food and take it to their mouth, the next requirement for successful BLW is having suitable *oral motor function* to eat pieces of whole food. It seems infants can eat “graspable” complementary whole foods that are soft in texture (e.g., a piece of cooked potato) without teeth [98]. The ability to chew a variety of foods with varying firmness and texture occurs alongside the eruption of teeth (seven months onwards) [98]. At six months of age the infant uses “a munching type of oral-motor activity, using up-and-down movements of the jaw” to break up food [99]. Furthermore they have developed lateral mobility in their tongue to move food around in the mouth and take food to the back of their mouth for swallowing [78]. Therefore it seems that most infants at six months possess the oral function to break up soft food in their mouth and move it around in order to swallow it. In fact it may be important that an infant is allowed the opportunity to use their oral skills as soon as they develop. Two studies [100,101] have demonstrated that infants exposed to textures after nine months of age were more likely to have feeding difficulties and be seen by their parents as fussy eaters compared with children who had been introduced to lumpier textures earlier. Although it is possible that some infants may have developed the oral motor function required to effectively, and safely, eat whole foods before six months of age, it would be unwise—and unnecessary—to attempt whole foods before six months because of the increased risks of inefficient feeding, and choking.

Anecdotally, the most commonly raised concern with BLW, and one that is shared by healthcare professionals and parents, is the *risk of choking* [8,17,22,102]. Choking is always a concern with young children and many of the choking episodes at this age are caused by food [103]. Choking is more likely when hard foods such as raw apple or round coin-shaped foods, including slices of sausage, are offered to children, or when the child is distracted while eating [104]. When complementary foods are being introduced, the infant is putting pieces of food into their mouth for the first time. This is a new experience requiring the coordination of chewing, swallowing and breathing. Whether children following BLW choke more than conventionally-fed children is unknown. We found in our recent qualitative study [22] that 30% of parents reported one or more episodes of choking with BLW. However, all parents who reported choking also reported that the infant independently dealt with the choking by expelling the food from their mouth through coughing, and that parents did not have to intervene with first aid. Parents who could recall the food that was responsible (*n *= 4/6) reported that raw apple was the food their infant had choked on. Brown *et al.* [17] also asked mothers about their choking experiences following BLW and found that mothers were initially concerned about choking but over time became less nervous as they were more able to distinguish between gagging (a safety mechanism that works by bringing large pieces of food forward for further chewing [105]) and actual choking. Gagging is very common among all infants and it can persist throughout infancy [106]. The place in the baby’s mouth at which the gag reflex is triggered moves back during the first year [78] so that generally children can eat finger foods with little or no gagging at about 8–9 months [87]. It has been argued that this is one of the advantages of BLW in that the BLW infant learns to eat finger foods at a time when the gag reflex very effectively keeps large pieces of food well to the front of the mouth, only allowing well masticated food to reach the back of the mouth for swallowing [7,8].

Obviously, not all handheld foods will be appropriate for self-feeding. In particular, parents must be advised to offer soft whole foods, and to avoid hard foods such as raw apple and nuts until later in childhood. The most commonly offered first foods (offered at six months of age) reported in our earlier work were vegetables (steamed or boiled pumpkin, potato, kumara (New Zealand sweet potato), broccoli, carrot) all of which can be cooked appropriately, *i.e.*, cooked until soft, and fruit (avocado, banana) [22]. It is also essential that children are sitting upright, and always have an adult present when they are eating. 

If the physical abilities are present to self-feed safely then the next question is whether a BLW diet provides *adequate nutrients*. A nutritionally adequate diet is essential for achieving optimal growth and development in the first year of life [4]. While breast milk provides sufficient nutrients for almost all healthy term infants to six months of age [107], it becomes increasingly difficult for an infant to get sufficient nutrients from breast milk alone after this time [108]. Therefore, once an infant reaches six months of age, complementary foods need to be introduced to meet the expanding nutrient requirements. This is the time when all infants should receive iron-rich complementary foods such as meat, meat alternatives or iron-fortified foods [3,4,109,110,111]. Spoon-feeding iron-fortified baby rice cereal is a popular way for parents to increase their infant’s iron intake and most infant cereals are fortified with iron to a level of 10–38 mg per 100 g of dry cereal [4]. However, infants following BLW are unlikely to eat infant cereals, given the semi-liquid form in which they are typically offered. Without this source of iron infants could be at increased risk of suboptimal iron status, which is already a concern for many infants [112]. Alternatively, because the infant following BLW is eating family foods there may be greater potential for a wider variety of iron-rich foods such as pieces of cooked beef, or liver, to be consumed. The bioavailability of iron in these foods is also much higher (~15.5%) than infant cereals (~3%) [113]. However parents following BLW may require clearer guidelines around the types and amounts of high iron foods to offer their infant in place of iron fortified infant cereal, both to ensure adequate intake, and to avoid choking. To date, no research has examined the food and nutrient intake of children following BLW to determine whether they are at increased risk of iron deficiency. The high iron requirements in this age group mean that BLW is not likely to be appropriate for children with delayed motor skills or oral motor function who would need to wait before they could self-feed effectively. Because it is essential that high iron foods are introduced from six months, it is not advisable to delay the introduction of complementary food beyond 180 days [3].

Similarly, the lack of dietary intake or growth information means it is not known whether BLW infants are meeting their *energy requirements*. It is feasible that lack of awareness of suitable BLW foods means some infants will be offered predominantly fruit and vegetables as the basis of their BLW diet which may provide insufficient energy for their needs. However, the opposite may also occur; where high energy, nutrient poor foods such as hot chips or chocolate biscuits that come in manageable sized pieces for infants to hold are viewed as suitable BLW foods by parents, although this is not recommended [7]. Examination of the dietary patterns of children following a BLW approach is urgently required. Some parents have reported concern about their infant’s energy intake at the beginning of BLW, although as the infant became more skilled and they realised that the infant was happy and healthy, this subsided [17]. 

As young children have sensitive appetite regulation skills [114], the energy intake of BLW infants should match their needs for growth provided they have the motor skills to feed themselves and are offered appropriate foods. In fact, allowing the infant to control their own food intake may lead to better self-regulation of energy intake and thus lower risk of overweight [17]. Although no reliable data exist on the energy needed by infants for adequate growth and physical activity, it is generally accepted that an infant who is growing within the accepted standards is in energy balance [115]. Only one study has evaluated the growth of infants following BLW compared with a (spoon-fed) control group [19]. They found that more of the spoon-fed infants were overweight or obese (8/63 *cf.* 1/63), and more of the BLW infants were underweight (3/63 *cf.* 0/63), but the number of cases was too small to allow statistical comparisons, and both observations require corroboration. 

One of the basic tenets of BLW is offering the infant *family foods* from the start of complementary feeding, but this may not always be suitable, depending on family food choice and the infant’s risk of allergy. Globally, many people eat a diet high in salt and sugar that should be avoided in young children. For those following the conventional method of feeding, commercial baby food or home-prepared purées don’t typically include added salt or sugar. One small pilot study (*n* = 10 infants) has examined the use of family food in those following BLW [20]. Unexpectedly, parents only offered their children 57% of the same foods they were consuming and the reasons for not offering the same foods were unclear. Practically speaking, there will be family foods that are suitable for the infant and many that require modification to reduce salt and sugar levels (for example casseroles flavoured with salt, stock, gravy or canned tomatoes). The alternative is for the family to modify their diet so that it is in keeping with the infant’s diet, which one study has shown may occur [17]. 

Family meals are also not necessarily appropriate for infants for whom there are allergy concerns. There is significant variation in national guidelines around what and how foods should be introduced to the infant at six months because of the risks of food allergy. Some national health advisory groups [4,116,117] recommend that all new foods should be introduced individually and gradually, whereas others [13] suggest that this only applies to the common “high risk” foods such as milk, egg, peanut, soy, nuts and wheat [118]. Moreover there seems to be a growing body of evidence that delaying the introduction of certain foods associated with the risk of allergy [118] such as cow’s milk, eggs, wheat, gluten, nuts, peanut products, seeds, and fish does not reduce the risk and may even increase the risk [62,119,120,121]. However, more stringent recommendations also exist in some countries, which even advise on the order in which to introduce specific foods [122]. In BLW, infants are allowed a range of family foods (apart from those carrying a choking risk) once they reach six months [7]. Although BLW encourages introducing a variety of foods, it does also emphasize that if there is a family history of allergy or a known or suspected digestive disorder then BLW should be discussed with the health advisor [7]. Introducing foods individually doesn’t reduce the risk of allergy but improves the likelihood of detecting reactions or sensitivities (e.g., intolerances) to foods. Given the current controversy surrounding allergies and the limited research on BLW we have no indication of whether BLW (*i.e.*, allowing the infant to eat family foods from six months) would have any effect on allergy risk. However it is reasonable to suppose that if the infant is exposed to family foods, especially mixed dishes, and an allergic reaction results, it may reduce the likelihood of identifying the specific food allergen that causes a reaction. 

In summary, although most infants probably have the skills to self-feed safely at six months, more research is needed to determine the nutrient intakes of infants following BLW and to ascertain whether parents need more guidance about appropriate foods to offer, both in terms of choking and nutrient adequacy. Baby-Led Weaning requires teamwork from the parent (to offer healthy and appropriate foods) and from the infant (to self-feed). This means it is also important that parents understand that a different approach may need to be taken for preterm infants or those with developmental delay, at least until they are able to effectively convey food to their mouth, and safely chew and swallow it, and also for those at increased risk of allergy; and perhaps during and following illness.

## 6. Can Parents Meet Expectations around Family Meals and Continued Breastfeeding?

Several studies have reported benefits of eating *family meals *together including healthier eating patterns and improved psychological well-being [123,124]. In BLW the expectation is that the infant shares all their meals with a family member. This is important primarily from a safety point of view, as infants must be watched when eating in case they choke, but is also likely to facilitate the child sharing the same food as the rest of the family, and may make prolonged self-feeding attempts while the infant learns these skills more manageable for parents. However, the family meal research, to date, has focused on children and adolescents and without research on the infant at the family meal we do not know either whether it is essential to include infants at all family meals, or the prevalence of infants being included at the family meal. Instead, studies that have observed infant mealtimes tend to only involve one parent (predominantly the mother) and use mealtimes as an opportunity to assess specific elements of parenting (e.g., parenting style or maternal control [125,126,127]) around feeding, or the impact of feeding technique (e.g., responsive feeding as mentioned earlier in this review [128,129,130]) on infant related outcomes. 

It has been suggested that the frequency of family meals may be declining [131,132], but data are mixed and the frequency of family meals seems to vary between countries (in Italy, 90% of families have been reported to be sharing meals together several times per week compared to 65% of American families [133]). Proposed reasons for a decline in the frequency of family meals have included changes in employment [131,133,134] and family structure [135,136]. For example, there has been an increase in the number of mothers employed full-time outside the home [137,138]. Families with a mother employed full-time outside the home appear to be 30% less likely to have regular family meals compared to those with a stay-at-home mother [133]. Although some research has shown a decline in family meals, others [139] have shown an increase in the number of families eating at home. One study in the USA reported that 52% of families in 2003 were sharing every meal together, but that this had increased to 73% in 2010 [139]. The inconsistencies in the family meal literature are likely due to differences in study design (e.g., using a retrospective design or conducting a single observation) and methodological issues (such as the definition of a family meal, *i.e.*, measuring eating together compared to eating at home, and measuring all meals compared to one meal per day). 

Family structure may affect the synchronization of family meals, with younger (12 years old) children reported to share more family meals than older (17 years old) children [136]. This may be due to older children being involved in more activities outside the home (e.g., work and sports) [140]. This is likely to vary from family to family, and some infants may be at home for meals whereas others may be in childcare. Although it is presumably safe for the infant to eat with their caregiver at childcare this is not considered a “family meal”. Furthermore, the coordination of meals with the infant, particularly in the first few months of eating (6–8 months of age), may be difficult due to their pattern of eating frequent small meals [141]. Infants because of their small stomachs tend to follow a pattern of multiple daily feedings, rather than following a traditional pattern of three structured meals and snacks, which may mean that the infant is not necessarily hungry at the time of the family meal. In addition, mealtime coordination will also depend on the infant’s sleep pattern [142]. Having said this, parents following BLW appear to be able to work around the infant’s schedule. In Brown and Lee [17], mothers following BLW reported that in some cases timing of meals was adapted to suit the infant’s pattern and when this was not feasible one parent would sit with the infant and eat a snack whilst the infant ate their meal. We have found [22] that parents following BLW had little difficulty achieving family meals together. Although mothers reported struggling with the concept of “baby eats what you eat”, they reported that their infant ate every meal with the family [22]. 

Sharing family meals potentially offers benefits for children of all ages, however without research specifically addressing family mealtimes during infancy we do not know if families are likely to achieve meals together, and indeed how important it is for the infant to be sharing family meals with the entire family if they are following BLW. Managing to coordinate family meals together with the infant may be difficult for some families to achieve and may require a substantial change in family practices, so it will not be achievable for all meals for all families. 

*Continued milk feeding* (preferably breastfeeding) on demand may also be particularly challenging for mothers who return to work. Although the WHO recommends that infants continue to be breastfed alongside complementary food until 2 years of age the research suggests that achieving the recommendation is uncommon in developed countries, with approximately one-quarter still breastfeeding at 12 months [80,81]. It is also uncertain whether this feeding is on demand from the infant, one of the tenets of BLW. 

However, if the demands upon families mean they do not comply with the expectations of having family meals together and continuing breastfeeding on demand then all is not lost. These expectations are likely to be less important for achieving the potential benefits associated with BLW than the other fundamental components of BLW such as the delayed introduction of complementary food to six months. 

## 7. Conclusion: What Questions Remain and How Feasible Is BLW as an Approach to Infant Feeding?

Although the prerequisites for BLW, including milk feeding (preferably exclusive breastfeeding) to six months and not starting complementary foods until six months, may be hard for some families to achieve because of certain social and psychosocial factors, they are not impossible. Very few mothers cannot physically breastfeed their children [143] and research has confirmed that most healthy full term infants do not need complementary foods until six months of age. Developmentally the majority of infants appear to be equipped for BLW at six months. It appears that most normal healthy infants will possess the gross motor skills and oral functioning needed to self-feed whole foods successfully and safely, provided that appropriate foods are offered by their parents. However, research is required on whether infants following BLW have adequate energy and iron intakes in particular. It is possible that such studies will identify a need for specific guidelines to address the energy content of the foods offered, and how often they should be offered (to avoid failure to thrive, or indeed obesity) as well as how BLW infants can meet their iron requirements (as iron fortified infant cereals are usually spoon-fed). This will need to be done whilst encouraging parents to offer culturally appropriate family foods. Guidelines are needed for feeding infants when they are unwell or recuperating. These guidelines will assist parents and early childhood centers who care for infants following BLW. It is not possible to comment on the appropriateness of BLW for infants in terms of food allergy in the absence of a consensus on the role of any type of complementary feeding in the development and identification of food allergy. 

The expectation that family meals will be eaten together may be somewhat difficult for families to achieve because it may require a substantial change in family practices. Continued milk feeding (preferably breast milk) on demand may also be particularly challenging for mothers who return to work. However, for families who want to follow BLW then these issues will not be insurmountable. Ultimately, the fundamentals of BLW (*i.e.*, self-feeding handheld food only) can be followed even in the absence of these practices—as long as there is careful adult supervision of infant eating, and as with conventional spoon-feeding, complementary foods are not expected to provide the majority of nutrient intake until the infant is at least 8 months of age.

The primary focus of infant feeding needs to continue to be responsive feeding, in particular, responding to infant hunger and satiety cues; being patient and encouraging the child to eat, but never forcing them; and experimenting with different food combinations, tastes, textures. In many ways, BLW provides a framework for infant feeding that encourages responsive feeding. Although BLW is probably achievable for most infants and their families, it may not be the best option for all infants at all times. Infants with developmental delay or other oral or motor problems would probably not do well following BLW. Furthermore, it remains to be seen whether infants following BLW are able to self-feed sufficiently while they are unwell or recuperating to be able to meet their energy and nutrient requirements without needing some assistance with eating.

Baby-Led Weaning appears to be a feasible option for introducing complementary foods for many infants and could conceivably have beneficial effects on the infant’s nutrition and development. 

However, many unanswered questions remain, including:

(1) Do parents who follow a BLW approach generally wait until the infant is six months of age before starting complementary foods? If not, do they use formula, introduce BLW early, or use purées until the child is able to self-feed?
(2) How is BLW defined; can a limited amount of spoon-feeding and purées be used and what commonly occurs in practice?
(3) Does responsive parenting lead to BLW or are people interested in BLW more likely to use responsive parenting practices?
(4) Do infants following BLW obtain sufficient nutrients, including energy and iron, or eat a more diverse range of foods?
(5) Is BLW a viable approach for obesity prevention, via improving self-regulation of energy intake?
(6) Are iron deficiency, choking and growth faltering real concerns for those following a Baby-Led approach?

Ultimately, the feasibility, benefits and risks of BLW as an approach to infant feeding can only be determined in a study in which infants and their families are randomized to following BLW, and their outcomes are compared to those of a control group following standard feeding practices. Given the popularity of BLW amongst parents, such a study is urgently needed.
